# *ARMC5* mutations in familial and sporadic primary bilateral macronodular adrenal hyperplasia

**DOI:** 10.1371/journal.pone.0191602

**Published:** 2018-01-25

**Authors:** Liping Yu, Junqing Zhang, Xiaohui Guo, Xiaoyu Chen, Zhisong He, Qun He

**Affiliations:** 1 Deparment of Endocrinology and Metabolism, Peking University First Hospital, Xicheng District, Beijing, China; 2 Department of Urology, Peking University First Hospital, Xicheng District, Beijing, China; NIDCR/NIH, UNITED STATES

## Abstract

To investigate *Armadillo repeat-containing 5* (*ARMC5*) mutations in Chinese patients with familial and sporadic primary bilateral macronodular adrenal hyperplasia (PBMAH), we performed clinical data collection and *ARMC5* sequencing for three PBMAH families and 23 sporadic PBMAH patients. *ARMC5* pathogenic germline mutations were identified in all 3 PBMAH families. Secondary *ARMC5* somatic mutations were found in two adrenal nodules from two PBMAH family members with *ARMC5* germline mutations. PBMAH family members with *ARMC5* pathogenic germline mutations displayed various clinical manifestations. *ARMC5* pathogenic germline mutations were identified in 5 sporadic PBMAH patients among whom one patient displayed both hypercortisolism and primary aldosteronism. We detected a total of 10 *ARMC5* pathogenic mutations, of which 8 had not been previously reported. Our results suggest that *ARMC5* pathogenic germline mutations are common in familial and sporadic Chinese PBMAH patients, and demonstrate the importance of *ARMC5* screening in PBMAH family members to detect patients with insidious PBMAH.

## 1. Introduction

Primary bilateral macronodular adrenal hyperplasia (PBMAH), also called ‘ACTH-independent macronodular adrenal hyperplasia (AIMAH)’, is a peculiar subtype of Cushing’s syndrome characterized by multiple benign nodules in bilateral adrenal cortexes. The prevalence of PBMAH was thought to be less than 2% among all cases of endogenous Cushing’s syndrome [1]. Due to the broad application of imaging technology, more PBMAH patients with mild hypercortisolism are identified, and the prevalence is higher than previously thought [1, 2]. PBMAH patients display insidious onset and slow progression of symptoms, leading to a late diagnosis of PBMAH between the ages of 40 and 70 years old, with a high proportion of subclinical Cushing’s syndrome [2–4].

Currently, the pathophysiology of PBMAH is not fully understood. Previous studies have indicated that aberrant G-protein-coupled membrane receptors, such as the gastric inhibitory polypeptide receptor [5], beta-adrenergic receptor [6], vasopressin receptor [7, 8], serotonin 4 receptor [9] and luteinizing hormone receptor [10], are expressed in the hyperplastic adrenal cells regulating cortisol synthesis. Recently, ACTH produced within the hyperplastic adrenal nodules was found to contribute to cortisol hypersecretion in PBMAH [11]. Germline and somatic mutations of several genes were also reported to be involved in the mechanisms causing PBMAH. Genes associated with PBMAH include *ARMC5* (*Armadillo repeat-containing 5*), *PDE11A* (*Phosphodiesterase 11A*) [12], *GNAS* (encoding the stimulatory G protein alpha subunit) [13, 14], *APC* (*Adenomatous polyposis coli*) [13], and *FH* (*Fumarate hydratase*) [13], among others.

*ARMC5* was found to be the most frequently mutated gene in PBMAH. A series of clinical genome sequencing studies revealed that the *ARMC5* mutation plays an important role in adrenal tumorigenesis in PBMAH. In 2013, *ARMC5* pathogenic germline mutations were first identified in 55% (18 out of 33) of PBMAH patients [15]. Subsequently, several studies reported damaging mutations of *ARMC5* in PBMAH patients with frequencies ranging from 19.6% to 26% [2, 16, 17]. Ten out of 13 familial cases exhibited *ARMC5* pathogenic germline mutations, suggesting that *ARMC5* is probably a major gene underlying familial PBMAH [18–23]. Among these PBMAH families, two were of Asian descent. One is a Chinese family with a germline missense mutation of *Endothelin receptor type A (EDNRA)*, while the other is a Japanese family with *ARMC5* germline deletion from exon1 to exon 5 [18, 22]. PBMAH family members with *ARMC5* germline mutations could display mild hypercortisolism or normal cortisol secretion without an obvious cushingoid appearance [19–21].

With respect to ARMC5 function, overexpression of non-mutated *ARMC5* in H295R and HeLa cells causes increased cell death, while mutated *ARMC5* does not have this effect, indicating that *ARMC5* likely functions as a tumor suppressor gene [15]. *ARMC5* somatic mutations have been detected in hyperplastic adrenocortical nodules of PBMAH patients in addition to the germline mutations, supporting a ‘two-hit’ model for *ARMC5* as a tumor suppressor gene [15]. Expression of *Armc5* mRNA is abundant in mouse adrenal glands. *Armc5* knockout mice exhibit adrenal hyperplasia as they age [24]. Moreover, *Armc5* also plays an important role in T-cell differentiation and apoptosis. *Armc5* knockout mice exhibit a defective immune response to lymphocytic choriomeningitis virus infection [24]. The signaling pathway in which ARMC5 is involved remains to be determined. However, yeast two-hybrid (Y2H) assays revealed that ARMC5 was able to bind 16 proteins several of which regulate cell apoptosis [24].

Little is known about the prevalence of *ARMC5* mutations in Chinese PBMAH families and sporadic patients. In this study, clinical data collection and *ARMC5* sequencing were carried out in 3 PBMAH families and 23 sporadic PBMAH patients in order to investigate *ARMC5* mutations in these candidates.

## 2. Subjects and methods

### 2.1 Participants

This study was approved by the Ethic Committee of Clinical Studies in Peking University First Hospital. The approval number is 2016 [1045]. This study included 23 sporadic PBMAH patients and 3 PBMAH patients with a family history of PBMAH who were treated at Peking University First Hospital between January 2008 and April 2016. Since May 2016, we retrospectively analyzed the patients’ clinical data and prospectively collected peripheral blood samples. We then collected the clinical data and blood samples from members of 3 PBMAH families. PBMAH family-1, family-2 and family-3 had 9, 7 and 6 members, respectively, who participated in the clinical data collection and gene sequencing. Adrenal nodule samples from four PBMAH patients (F1-II-1, F2-II-3, P-4 and P-7) were collected after the surgeries. Sporadic patients were defined as those without a family history based on the descriptions given by the patients. All participants signed informed consents. Each participant was identifiable during and after data collection. The individual in this manuscript has given written informed consent to publish these case details.

### 2.2 Hormonal measurements and diagnostic criteria

Serum cortisol, urinary cortisol and plasma ACTH levels were measured using an electrochemiluminescence immunoassay on a Roche Cobas e601 machine. Peripheral venous blood was taken at 08:00, 16:00 and 00:00 to determine the cortisol/ACTH secreting rhythm. In the peripheral venous blood, the normal range of serum cortisol levels at 8 am was 4.4 μg/dl to 19.9 μg/dl, while the normal range of plasma ACTH levels at 8 am was 7.2 PG/ml to 63.3 PG/ml. The normal range of 24-hour urinary free cortisol (24 h UFC) was 100 μg to 379 μg. An overnight dexamethasone suppression test (ODST) was conducted by administering 1 mg of dexamethasone at midnight. A low-dose dexamethasone suppression test (LDDST) was conducted by administering 0.5 mg of dexamethasone every 6 hours for two days. A high-dose dexamethasone suppression test (HDDST) was conducted by administering 2 mg of dexamethasone every 6 hours for two days. 24 h UFC and morning serum cortisol levels were measured throughout the LDDST and HDDST. A diagnosis of overt Cushing’s syndrome was defined as morning serum cortisol levels higher than 1.8 μg/dl after the LDDST with classical physical signs of Cushing’s syndrome [25]. Subclinical Cushing’s syndrome was defined as morning serum cortisol levels higher than 1.8 μg/dl after the ODST plus elevated 24 h UFC or suppressed plasma ACTH (<10 pg/ml), without classical physical signs of Cushing’s syndrome [26].

Screening for primary aldosteronism (PA) was conducted in suspected patients according to the Endocrine Society guidelines. Before testing for aldosterone and renin levels, the patients discontinued beta-blockers, angiotensin-converting enzyme inhibitors and angiotensin Ⅱ receptor blockers for at least 3 weeks; diuretics for 6 weeks; and spironolactone for 8 weeks. The aldosterone-to-renin ratio (ARR) was calculated as the plasma aldosterone concentration (PAC, ng/dl) divided by the plasma renin activity (PRA, ng/ml/h). Peripheral venous blood was collected at 6 am after the patients had been lying down for at least 6 hours in order to test the supine PAC and PRA levels. The patients then stood up for 2 hours, until 8 am, to test the upright PAC and PRA levels. A captopril challenge test was conducted by administering 50 mg of captopril orally to patients after the patients had been sitting for at least 1 hour. Blood samples for measuring PAC and PRA were collected at Time 0 and at 2 hours after captopril was administered. Suppression of the captopril challenge test was defined as a decrease in PAC levels of at least 30% at 2 hours after captopril was administered compared to PAC levels at Time 0. PA was diagnosed by a recumbent PAC of more than 15 ng/dl, an ARR of more than 20, and a non-suppressed captopril challenge test result [27].

### 2.3 Calculation of adrenal size

The volume of an adrenal nodule was calculated by the following equation: (π*a*b*c)/6, where ‘a’, ‘b’, and ‘c’ are the diameters of the nodule at the transverse axis, the sagittal axis and the vertical axis, respectively [4]. The size of a unilateral adrenal mass was the sum of the volumes of the nodules at a unilateral adrenal side.

### 2.4 DNA sequencing and analysis

DNA extraction from peripheral blood leukocytes and adrenal tissues was performed according to the manufacturer’s protocols (BIOMED from China and QIAGEN from Germany, respectively). *ARMC5* exonic sequences and the surrounding intronic sequences were amplified using previously described primers [15]. *ARMC5* was Sanger sequenced. The sequences were analyzed using several software programs including Bioedit, Mutation surveyor and DNAstar. The mutations were verified using the in silico software programs Mutation Taster (http://www.mutationtaster.org/), Polyphen-2 (http://genetics.bwh.harvard.edu/pph2/) [28] and SIFT (http://sift.jcvi.org/www/SIFT_enst_submit.html) [29]. The frequencies of the mutations were checked using ExAC (http://exac.broadinstitute.org/). All pathogenic mutations were tested twice in two independent experiments. The nomenclature of the variants was based on the *ARMC5* NCBI Reference Sequence NM_001105247.1 and was verified using the Mutalyzer program (http://www.LOVD.nl/mutalyzer/).

## 3. Results

### 3.1 Clinical characteristics and *ARMC5* mutations in PBMAH family-1

**Fig 1:**
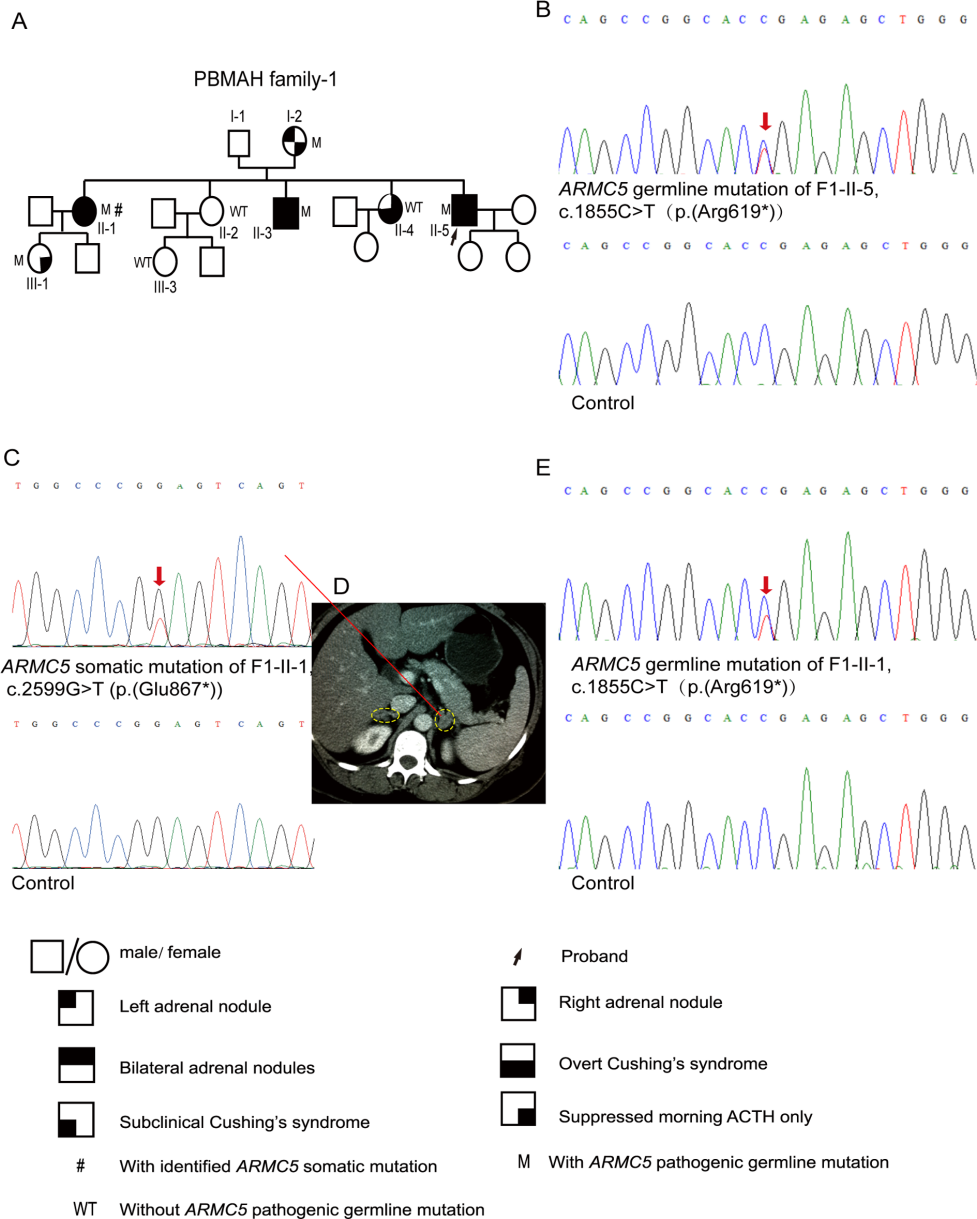
Pedigree of and *ARMC5* mutations in PBMAH family-1. A, Pedigree of PBMAH family-1. B, The *ARMC5* pathogenic germline mutation c.1855C>T (p.(Arg619*)) was detected in F1-II-5, the proband of PBMAH family-1. C, The secondary *ARMC5* somatic mutation c.2599G>T (p.(Glu867*)) was detected in an adrenal nodule from F1-II-1. D, The adrenal CT scan of F1-II-1. The adrenal glands are circled by dashed lines. E, The *ARMC5* germline mutation in F1-II-1. ‘M’ indicates family members with *ARMC5* pathogenic germline mutations, whereas ‘WT’ indicates those without *ARMC5* pathogenic germline mutations. The red arrows indicate the mutated sites in *ARMC5* sequences. Pedigree numbers were not assigned to the family members who were not available for the study.

PBMAH family-1 had 14 members including 9 who participated in the clinical data collection and gene sequencing (Fig 1A; Table 1). The proband (II-5 in family-1 or F1-II-5) was admitted to the hospital at 36 years old due to hypertension, diabetes and a cushingoid appearance including moon face, plethora and central obesity. The plasma ACTH levels of F1-II-5 were suppressed, and his 24 h UFC was as high as 4765 μg. The results of both the LDDST and the HDDST were not suppressed, and an adrenal CT scan revealed multiple nodules in the bilateral enlarged adrenal glands, suggesting the diagnosis of PBMAH. He underwent left adrenalectomy. One year after the surgery, the patient was admitted to the hospital again for a systemic evaluation. The ACTH levels were still suppressed, but the 24 h UFC was only 188 μg, which was much lower than before. Meanwhile, there were great improvements in blood pressure, glucose metabolism and the cushingoid appearance.

**Table 1:** Clinical data for PBMAH family members.

Family member	Gender	Age (y)	Morning cortisol (μg/dl)	Morning ACTH (PG/ml)	Adrenal masses and the size of unilateral adrenal masses	Diabetes	HT	Cushing’s syndrome	Adrenal surgery
Members of PBMAH family-1 with *ARMC5* pathogenic germline mutations									
F1-I-2	F	74	6.8	5.94	L: a nodule 0.5 cm in diameter; R: enlarged	IGT	Yes	No	No
F1-II-1	F	51	7.14	<1	L: multiple nodules, 4.4 cm^3;^ R:multiple nodules, 2.31 cm^3^	IGT	Yes	Yes, overt	Left
F1-II-3	M	49	17.89	<1	L: multiple nodules, 22.45 cm^3^ R: multiple nodules, 14.13 cm^3^	Yes	Yes	Yes, overt	No
F1-II-5 proband	M	36	25.33	<1	L: multiple nodules, 40.90 cm^3^; R: multiple nodules, 40.89 cm^3^	Yes	Yes	Yes, overt	Left
F1-III-1	F	32	5.84	6.97	Normal	No	No	No	No
Members of PBMAH family-1 without *ARMC5* pathogenic germline mutations									
F1-I-1	M	76	13.49	35.41	Normal	Yes	Yes	No	No
F1-II-2	F	51	10.6	12.31	Normal	No	No	No	No
F1-II-4	F	43	5.67	<1	L: Normal; R: a nodule 1.2 cm in diameter	IGT	Yes	Yes, overt	Right
F1-III-3	F	23	14.22	16.39	Normal	No	No	No	No
Members of PBMAH family-2 with *ARMC5* pathogenic germline mutations									
F2-II-1	M	53	16.78	11.45	L: a nodule, 4.19 cm^3^	No	No	No	No
F2-II-2 proband	M	43	23.7	<1	L: multiple nodules, 83.34 cm^3;^ R: multiple nodules	Yes	Yes	Yes, overt	Bilateral
F2-II-3	M	46	32.7	<1	L: multiple nodules; R: multiple nodules, 71.38 cm^3^	Yes	Yes	Yes, overt	Bilateral
F2-III-3	F	23	12.79	10.96	Normal	No	No	No	No
Members of PBMAH family-2 without *ARMC5* pathogenic germline mutations									
F2-I-2	F	74	9.98	14.7	Normal	No	No	No	No
F2-II-4	F	44	10.66	22.25	Normal	No	No	No	No
F2-III-1	F	18	18.49	16.09	Normal	No	No	No	No
Members of PBMAH family-3 with *ARMC5* pathogenic germline mutations									
F3-II-1	M	53	25.5	<1	Bilateral adrenal nodules;	Yes	Yes	Yes, overt	Bilateral
F3-II-2 proband	M	55	15.41	10.31	L: multiple nodules, 0.72 cm^3^; R: multiple nodules, 6.80 cm^3^	No	No	Yes, subclinical	Bilateral
F3-III-2	F	29	11.52	15.55	Normal	No	No	No	No
Members of PBMAH family-3 without *ARMC5* pathogenic germline mutations									
F3-II-3	F	52	8.96	9.35	Normal	No	No	No	No
F3-III-3	M	28	16.97	22.26	Normal	No	No	No	No
F3-III-4	F	26	15.9	12.09	Normal	No	No	No	No

IGT: impaired glucose tolerance; HT: hypertension.

Considering that the family history revealed that the proband’s two sisters also had hypertension and a cushingoid appearance, clinical screening for PBMAH was carried out in the members of PBMAH family-1 (Fig 1A; Table 1). F1-I-2, at 74 years of age, exhibited mildly suppressed morning ACTH and a small adrenal mass on the left side. F1-II-1 and F1-II-3, who were 51 and 49 years old, respectively, both exhibited overt Cushing’s syndrome and multiple bilateral adrenal nodules. F1-II-1 underwent left adrenalectomy, and the pathology of the operated adrenal was macronodular adrenal hyperplasia. F1-II-4, who was 43 years old, presented with overt Cushing’s syndrome and a mass in the right adrenal cortex. Surgery for removing the adrenal mass was conducted for F1-II-4, and the pathology of the surgically removed unilateral adrenal mass appeared to be adrenal adenoma. F1-III-1, at 32 years of age, exhibited mildly suppressed morning ACTH without obvious radiological abnormalities in the adrenal cortex. F1-II-2 and F1-III-3 had normal serum cortisol and ACTH levels as well as normal adrenal images (Fig 1A).

The *ARMC5* nonsense mutation c.1855C>T (p.(Arg619*)) was identified in F1-I-2, F1-II-1, F1-II-3, F1-II-5 and F1-III-1 (Fig 1B and 1E; S1A, S1B and S1C Fig). In addition to the germline mutation, a somatic *ARMC5* mutation, c.2599G>T (p.(Glu867*)), was detected in one of the excised adrenal nodules from F1-II-1 (Fig 1C and 1D).

### 3.2 Clinical characteristics and *ARMC5* mutations in PBMAH family-2

**Fig 2:**
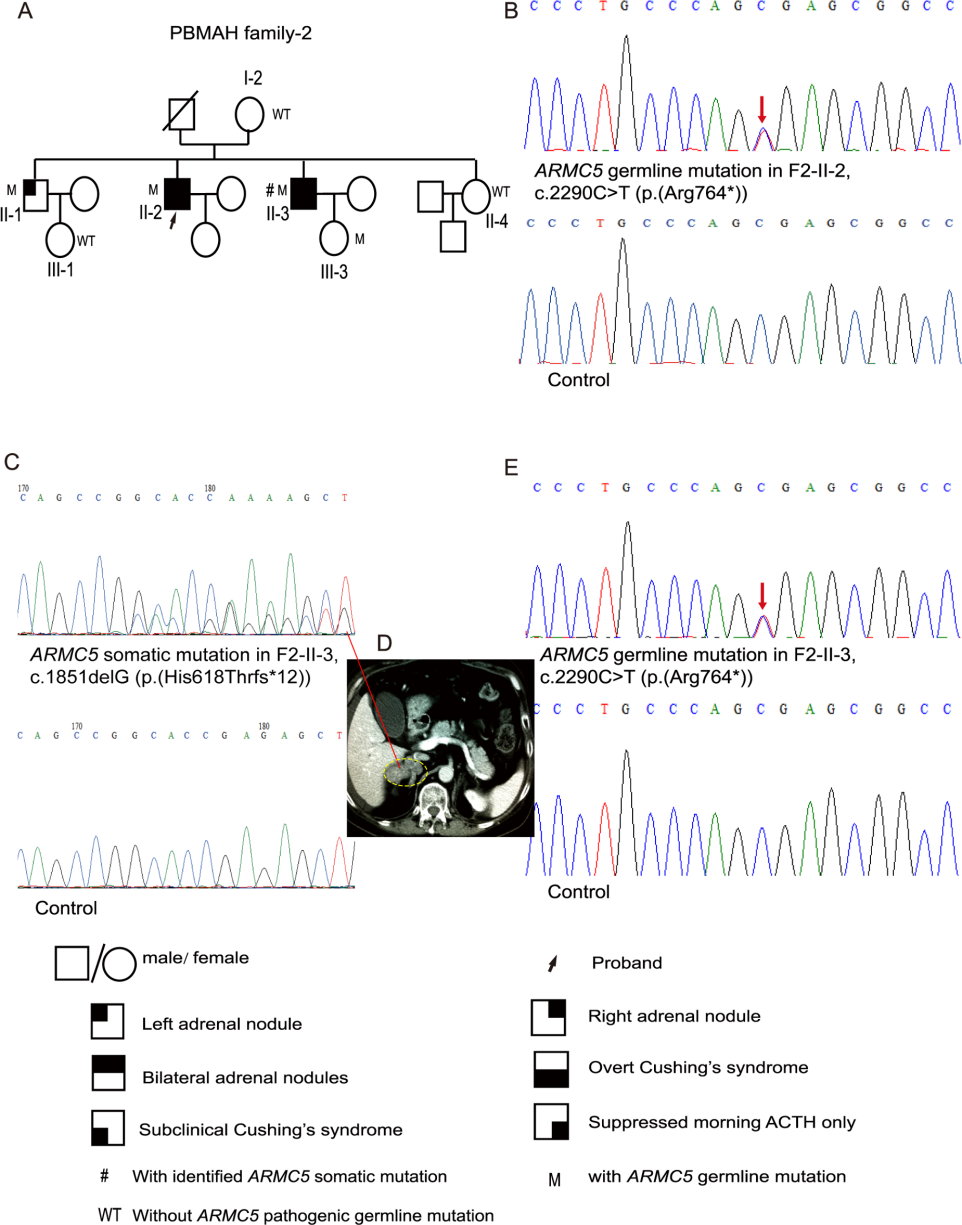
Pedigree of and *ARMC5* mutations in PBMAH family-2. A, Pedigree of PBMAH family-2. B, The *ARMC5* pathogenic germline mutation c.2290C>T (p.(Arg764*)) was detected in F2-II-2, the proband of PBMAH family-2. C, The secondary *ARMC5* somatic mutation c.1851delG (p.(His618Thrfs*12)) was detected in an adrenal nodule from F2-II-3. D, The adrenal CT scanning of F2-II-3. The right adrenal gland is circled by a dashed line. E, *ARMC5* germline mutation in F2-II-3. ‘M’ indicates family members with *ARMC5* pathogenic germline mutations, whereas ‘WT’ indicates those without *ARMC5* germline mutations. The red arrow indicates the mutated site in the *ARMC5* sequence. Pedigree numbers were not assigned to the family members who were not available in the study.

There were 9 members in PBMAH family-2, among whom 7 participated in the clinical data collection and gene sequencing (Fig 2A; Table 1). The proband (F2-II-2) was admitted to the hospital at 43 years of age due to hypertension, diabetes, and a cushingoid appearance including moon face, plethora, buffalo hump, striae, thin skin and central obesity. He had an abnormal serum cortisol rhythm and suppressed plasma ACTH levels. Further examinations indicated that he had osteoporosis, hypokalemia, dyslipidemia and ventricular hypertrophy in addition to hypertension and diabetes. A CT scan revealed multiple bilateral adrenal nodules. He underwent left adrenalectomy at first and partial right adrenalectomy half a year later. Afterwards, he took prednisone orally for replacement therapy with the dosage gradually adjusted to 5 mg in the morning and 2.5 mg in the afternoon. The symptoms, signs and complications caused by hypercortisolism were greatly improved after the surgeries on the bilateral adrenals.

The proband’s younger brother (F2-II-3) was admitted to the hospital at the age of 44 due to hypertension, diabetes, coronary heart disease and an apparent cushingoid appearance similar to his brother, the proband. He also suffered from severe osteoporosis, hypokalemia and dyslipidemia. He had overt Cushing’s syndrome and multiple bilateral adrenal nodules, indicating the diagnosis of PBMAH (Table 1). This patient first underwent left adrenalectomy. Afterwards, his blood pressure, glucose metabolism and the cushingoid appearance were transiently improved. However, one and a half years after the left adrenalectomy, the cushingoid appearance returned, and his blood pressure and glucose levels were out of control. Thus, he underwent a right adrenalectomy, after which he took prednisone for replacement therapy with the dosage eventually adjusted to 5 mg in the morning and 2.5 mg in the afternoon. After bilateral adrenalectomy, his appearance returned to normal, and his blood pressure and glucose levels were able to be controlled.

Clinical screening was further carried out for other members of PBMAH family-2. The proband’s elder brother (F2-II-1) presented with only a unilateral adrenal mass detected by adrenal imaging (Table 1). F2-I-2, F2-II-4, F2-III-1 and F2-III-3 all had normal adrenal images. F2-I-2, F2-II-1, F2-III-1 and F2-III-3 did not have excessive cortisol secretion and presented no signs or symptoms associated with hypercortisolism (Fig 2A; Table 1).

The *ARMC5* nonsense mutation c.2290C>T (p.(Arg764*)) was detected in F2-II-1, F2-II-2, F2-II-3 and F2-III-3 (Fig 2B and 2E; S2A and S2B Fig). In addition to the germline mutation, a secondary somatic *ARMC5* alteration, c.1851delG (p.(His618Thrfs*12)), was identified through sequencing of the DNA available from an adrenal nodule of F2-II-3 (Fig 2C and 2D).

### 3.3 Clinical characteristics and *ARMC5* mutations in PBMAH family-3

**Fig 3:**
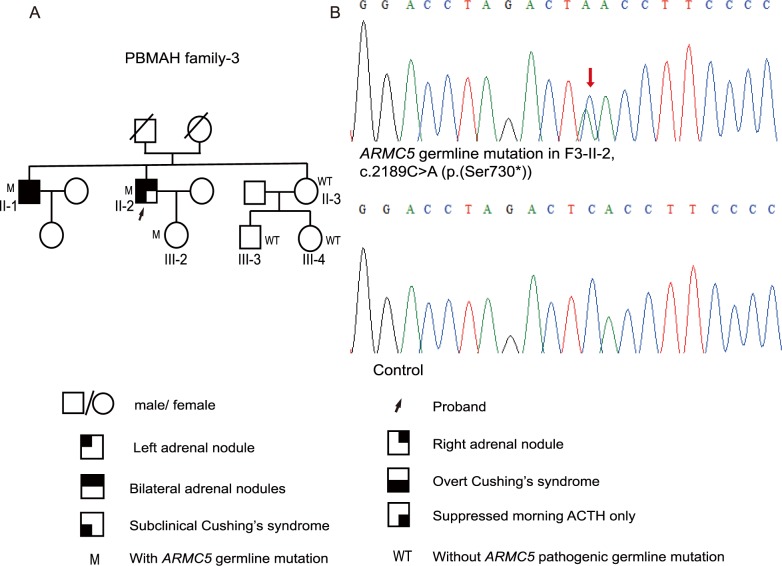
Pedigree of and *ARMC5* germline mutation in PBMAH family-3. A, Pedigree of PBMAH family-3. B, The *ARMC5* pathogenic germline mutation c.2189C>A (p.(Ser730*)) was detected in F3-II-2. ‘M’ indicates family members with *ARMC5* pathogenic germline mutations, whereas ‘WT’ indicates those without *ARMC5* pathogenic germline mutations. The red arrow indicates the mutated site in the *ARMC5* sequence. Pedigree numbers were not assigned to he family members who were not available for the study.

There were 7 members in PBMAH family-3. Six of them participated in the clinical data collection and gene sequencing (Fig 3A; Table 1). Adrenal masses were incidentally detected in a thoracic CT scan of the proband (F3-II-2, age 55). He then had an adrenal contrast CT scan, and multiple bilateral adrenal nodules were found. The 24 h UFC exceeded twice the upper limit of the normal range. Based on a query of the family history of the proband, we learned that his brother (F3-II-1) was diagnosed with PBMAH at age 53. F3-II-1 had typical symptoms and signs due to excess cortisol levels. He underwent bilateral adrenal excision. Afterwards, he took hydrocortisone tablets for replacement therapy with the dosage eventually adjusted to 20 mg in the morning and 10 mg in the afternoon. His symptoms, signs and complications caused by hypercortisolism were greatly improved after bilateral adrenalectomy. F3-II-3, F3-III-2, F3-III-3 and F3-III-4 had no signs or symptoms of hypercortisolism (Fig 3A; Table 1).

*ARMC5* was sequenced in F3-II-1, F3-II-2, F3-II-3, F3-III-2, F3-III-3 and F3-III-4. The nonsense mutation c.2189C>A (p.(Ser730*)) was detected in F3-II-1, F3-II-2 and F3-III-2 (Fig 3B; S3A Fig). No *ARMC5* pathogenic mutation was found in F3-II-3, F3-III-3 and F3-III-4.

### 3.4 *ARMC5* mutations in sporadic PBMAH patients

Five out of 23 sporadic PBMAH patients (21.7%) had *ARMC5* pathogenic germline mutations. The five mutations were c.1214delG (p.(Gly405Alafs*56)), c.318delG (p.(Ser107Argfs*30)), c.2564delT (p.(Val855Glyfs*62)), c.622_623insC (p.(Gln208Profs*15)) and c.523delG (p.(Ala175Profs*7)) (S4 Fig). These frameshift mutations have not been described in previous studies. Adrenal tissues from two sporadic PBMAH patients without germline *ARMC5* pathogenic mutations, P-4 and P-7, were available. *ARMC5* was amplified and sequenced but no *ARMC5* pathogenic mutation was identified in the adrenal tissues from these two patients.

Interestingly, among the five sporadic PBMAH patients with *ARMC5* germline mutations, P-15 was diagnosed with not only Cushing’s syndrome but also PA, while the other four patients had only Cushing’s syndrome (Table 2). P-15 was admitted to the hospital because of refractory hypertension and hypokalemia. Four kinds of anti-hypertension medications, benidipine, arotinolol, telmisartan and compound amiloride hydrochlorothiazide tablets were prescribed, but his blood pressure was still approximately 150/100 mmHg. The supine and upright PACs were as high as 19 ng/dl and 24 ng/dl, respectively, while the supine and upright plasma renin activities were as low as 0.08 ng/ml/h and 1 ng/ml/h, respectively. The results of the captopril test were not suppressed, suggesting the diagnosis of PA. P-15 underwent a unilateral adrenalectomy, after which his blood pressure was easily controlled and his plasma potassium concentration became normal. Histologically, the resected adrenal was comprised of mainly clear-type cells that were rich in lipids (S5 Fig).

**Table 2:** Clinical data of sporadic PBMAH patients with *ARMC5* pathogenic germline mutations.

PBMAH patients	Gender	Age (y)	Morning cortisol (μg/dl)	Morning ACTH (PG/ml)	Adrenal masses and the size of unilateral adrenal masses	Diabetes	HT	PA	Cushing’s syndrome	Adrenal surgery
P-3	F	60	11.53	<1	R: enlarged, no obvious nodules; L: multiple nodules, 5.23 cm^3^	Yes	Yes	No	Yes, overt	No
P-6	F	52	59.96	<1	Bilateral adrenal nodules	Yes	Yes	No	Yes, overt	Unilateral
P-8	M	61	23.57	<1	L: multiple nodules, 63.14 cm^3^; R: multiple nodules, 75.00 cm^3^	No	Yes	No	Yes, overt	Bilateral
P-15	M	54	19.76	2.21	Bilateral adrenal nodules	IGT	Yes	Yes	Yes, overt	Unilateral
P-22	M	68	21.2	15.92	L: multiple nodules, 15.54 cm^3^; R: a nodule, 0.27 cm^3^	Yes	Yes	No	Yes, subclinical	No

IGT: impaired glucose tolerance; HT: hypertension; PA: primary aldosteronism.

All 23 sporadic PBMAH patients exhibited multiple bilateral adrenal nodules with subclinical or overt Cushing’s syndrome. Comparing the clinical features of the *ARMC5-*mutated sporadic PBMAH patients with those of the *ARMC5*-wildtype sporadic PBMAH patients, there were no significant differences in age, gender, BMI, UFC levels, or cortisol levels before or after the LDDST. Additionally, there were no significant differences in the percentages of hypertension, diabetes, PA, or overt Cushing’s syndrome (S1 and S2 Tables).

## 4. Discussion

In this study, we detected a total of 8 *ARMC5* pathogenic germline mutations and 2 *ARMC5* pathogenic somatic mutations (Figs 1, 2 and 3; S4 and S6 Figs). Among these 10 mutations, c.1855C>T (p.(Arg619*)) and c.2290C>T (p.(Arg764*)) were identified in two PBMAH patients in a previous study, and only p.(Arg619*) was found in ExAC with a frequency of 0.00002695 [2], whereas the other 8 *ARMC5* mutations have not been reported before.

Various phenotypes were displayed in PBMAH family members with *ARMC5* pathogenic mutations. Some presented with subclinical hypercortisolism (F3-II-2), some exhibited mildly suppressed ACTH (F1-I-2 and F1-III-1), and others exhibited normal cortisol secretion (F2-II-1, F2-III-3 and F3-III-2). Regarding adrenal imaging, some family members with *ARMC5* pathogenic mutations presented with bilateral adrenal masses, some had unilateral adrenal masses, and others had normal adrenals. The ‘two-hit’ theory of *ARMC5* is considered the most likely underlying cause of the various phenotypes observed in PBMAH family members with *ARMC5* pathogenic mutations. A previous study reported that the somatic mutation and the germline mutation were close to each other in two tumor tissues. This study then cloned the PCR products using the pGEM-T Easy vectors and DH-5 bacteria [15]. Sequences of the clones indicated that the somatic mutation and the germline mutation were on two different alleles, supporting the ‘two-hit’ theory of *ARMC5* [15]. In our study, the somatic mutations and the germline mutations were located on different exons distant from one another. Thus it is difficult for us to conduct a similar cloning experiment. Knudson’s ‘two-hit’ theory is that, in a hereditary tumor disorder, the phenotype will occur when there is a secondary somatic mutation in addition to the germline heterozygous mutation [30]. Thus, if the secondary somatic mutation arises later or never occurs in PBMAH family members with *ARMC5* germline mutations, the enlargement of the adrenals may begin at a later age or may never occur, thereby explaining the various manifestations in these family members with *ARMC5* germline mutations. Several *ARMC5*-mutated family members exhibited mild hypercortisolism or normal cortisol secretion; therefore, *ARMC5* gene screening can help identify those insidious patients. Moreover, these *ARMC5*-mutated family members without obvious clinical manifestations should be followed up throughout their lifetime because the phenotype may progress to overt hypercortisolism and the adrenals may grow larger in the future.

It is notable that F1-II-4 had Cushing’s syndrome and a unilateral adrenal mass but no *ARMC5* pathogenic germline mutation. We collected her blood samples twice and conducted *ARMC5* sequencing in two separate experiments, but no *ARMC5* pathogenic mutation was found. The CT image of F1-II-4 (S7B Fig) showed only one nodule on the right side, while her brother, F1-II-5, had multiple adrenal nodules (S7A Fig). Histologically, the cells of the resected nodule of F1-II-4 (S7D Fig) were less compact than those of F1-II-5 (S7C Fig). In a previous study, a PBMAH family member also presented with suppressed plasma ACTH levels and a unilateral adrenal mass without an *ARMC5* pathogenic germline mutation, although other family members with bilateral adrenal masses did have *ARMC5* germline mutations [20]. Perhaps the unilateral adrenal mass of F1-II-4 is just a typical adrenal adenoma with a different pathogenic mechanism from the bilateral adrenal masses of her siblings who carried *ARMC5* pathogenic germline mutations.

The *ARMC5* germline alterations c.1214delG (p.(Gly405Alafs*56)), c.318delG (p.(Ser107Argfs*30)), c.2564delT (p.(Val855Glyfs*62)), c.622_623insC (p.(Gln208Profs*15)) and c.523delG (p.(Ala175Profs*7)) were identified in 5 out of the 23 (21.7%) sporadic PBMAH patients (S4 and S6 Figs). These *ARMC5* germline alterations have never been reported in previous studies. Previous studies revealed that the percentage of *ARMC5* germline mutations in sporadic PBMAH patients ranged from 19.6% to 55% [2, 15–17]. A recent study screened *ARMC5* mutations in 8 first-degree relatives of 3 sporadic *ARMC5*-mutated PBMAH patients and detected 4 carriers of *ARMC5* germline mutations [17]. Given that the onset of PBMAH is often after 40 years of age and that a high proportion of PBMAH patients exhibited subclinical hypercortisolism or only mildly suppressed ACTH levels, as indicated in our study and in several previous studies [2, 16, 19, 21, 23], the family histories of so-called sporadic PBMAH patients may be unknown. Therefore, it is necessary to conduct *ARMC5* sequencing for the family members of so-called sporadic PBMAH patients who possess *ARMC5* germline mutations.

We identified an *ARMC5* pathogenic germline mutation in a patient (P-15) who presented with both Cushing’s syndrome and PA. In previous studies, *ARMC5* pathogenic germline mutations were identified in six PA patients who were all African Americans [31], whereas no *ARMC5* pathogenic germline mutations were detected in Caucasian PA patients [31, 32]. Additional sequencing of *ARMC5* in more Asian PA patients is needed to test whether *ARMC5* is associated with PA in the Asian subpopulations. The mechanism by which *ARMC5* is involved in PA is not yet clear. However, *CTNNB1*, which is also an ARM-containing gene that encodes beta-catenin, is a causative gene for PA [33]. The WNT/beta-catenin signaling pathway potentially regulates aldosterone synthesis [33]. *ARMC5* contains tandem ARM repeats similar to *CRNNB1*, but it is unknown whether *ARMC5* is associated with the WNT pathway. Further studies are necessary to clarify the mechanism by which *ARMC5* mutations affect aldosterone secretion.

In our study, four out of five mutations in sporadic PBMAH patients were within or adjacent to the ARM repeat domains in *ARMC5*, whereas two out of three mutations in PBMAH families were within or adjacent to the BTB/POZ (broad complex Tramtrack bric-a-brac/ Pox virus and zinc finger) domain. To explore the association between the mutation sites and the two specific structures, we summarized the *ARMC5* germline mutations of sporadic PBMAH patients and PBMAH families reported in previous studies (S8 Fig). More than 60% of the mutations are within or adjacent to the ARM-repeat domains and the BTB/POZ domain. The ARM-repeat domains and the BTB/POZ domain are crucial for the function of their host proteins. The ARM-repeat structure binds to interaction partners, regulating adrenal cortex development [34], T cell development [24, 35], bone formation [36], and tumor suppression [34]. Several proteins containing BTB/POZ domains are recognized as transcriptional regulators of tumor suppression [37], T-cell proliferation, and organ development [38]. The importance of ARM-repeat domains and BTB/POZ domains suggests that mutations around or within these domains of *ARMC5* affect the function of ARMC5. A previous study reported that ARMC5 was associated with adrenal cell apoptosis, T-cell differentiation and proliferation, and immune response [24]. Using a yeast two-hybrid (Y2H) assay, ARMC5 was shown to bind 16 proteins [24], but the signaling pathways in which ARMC5 is involved remain unclear.

In conclusion, our results suggest that *ARMC5* pathogenic germline mutations are common in Chinese PBMAH families and sporadic PBMAH patients. Our results demonstrate the importance of *ARMC5* screening for PBMAH family members to detect insidious PBMAH patients.

## Supporting information

S1 Fig The *ARMC5* sequences around the mutated site in other members of PBMAH family-1. F1-I-2, F1-II-3 and F1-III-1 exhibited the *ARMC5* point mutation c.1855C>T as indicated in panels A, B and C. F1-I-1, F1-II-2, F1-II-4 and F1-III-3 did not exhibit any *ARMC5* mutation in the sequences presented in panels D, E, F and G.
(PDF)

S2 Fig The *ARMC5* sequences around the mutated site in other members of PBMAH family-2. F2-II-1 and F1-II-3 exhibited the *ARMC5* point mutation c.2290C>T as indicated in panels A and B. F2-I-2, F2-II-4 and F2-III-1 did not exhibie any *ARMC5* mutation in the sequences presented in panels C, D and E.
(PDF)

S3 Fig The *ARMC5* sequences around the mutated site in other members of PBMAH family-3. F3-II-1 and F3-III-2 exhibited the *ARMC5* point mutation c.2189C>T as indicated in panels A and B. F3-II-3, F3-III-3 and F3-III-4 did not exhibit any *ARMC5* mutation in the sequences presented in panels C, D and E.
(PDF)

S4 Fig Germline mutations of *ARMC5* in sporadic PBMAH patients. A, *ARMC5* frameshift mutation in P-3: c1214delG, p.(Gly405Alafs*56). B, *ARMC5* frameshift mutation in P-6: c.318delG, p.(Ser107Argfs*30). C, *ARMC5* frameshift mutation in P-8: c.2564delT, p.(Val855Glyfs*62). This mutation is indicated in the antisense sequencing graph. D, *ARMC5* frameshift mutation in P-15: c.622_623insC, p.(Gln208Profs*15). E, *ARMC5* frameshift mutation in P-22: p.(Ala175Profs*7).
(PDF)

S5 Fig The CT scanning image of the adrenal glands and the histological image of the resected adrenal gland of P-15. A. Multiple adrenal nodules were detected in the CT image. The red arrow indicates the right adrenal gland that was surgically resected. B. Histologically, the resected adrenal gland comprised primarily clear-type cells that were rich in lipid.
(PDF)

S6 Fig The distribution of *ARMC5* germline and somatic mutations identified in this study. The *ARMC5* protein contains 935 amino acids. The light gray squares indicate the Armadillo repeats, whereas the black square indicates the BTB/POZ domain. The germline mutations are indicated at the upper side of the peptide structure. The somatic mutations are presented under the peptide structure.
(PDF)

S7 Fig The CT scanning images of the adrenal glands of F1-II-5 and F1-II-4, and the histological images of the resected adrenal nodules of F1-II-5 and F1-II-4. A. The CT image of the adrenal glands of F1-II-5. The blue arrow indicates the left adrenal gland, which contained multiple nodules and was later resected by surgery. B. The CT image of the adrenal glands of F1-II-4. The red arrow indicates the only nodule on the right adrenal gland. C. The histological image of the resected adrenal nodules of F1-II-5. D. The histological image of the resected adrenal nodule of F1-II-4.
(PDF)

S8 Fig The distribution of *ARMC5* germline mutations identified in this and previous studies. The *ARMC5* protein contains 935 amino acids. The light gray squares indicate the Armadillo repeats, whereas the black square indicates the BTB/POZ domain. The germline mutations of PBMAH families are indicated at the upper side of the peptide structure. The germline mutations of PBMAH sporadic patients are indicated under the peptide structure.
(PDF)

S1 Table Clinical data of sporadic PBMAH patients without *ARMC5* pathogenic germline mutations. (DOCX)

S2 Table Comparison between sporadic PBMAH patients with and without *ARMC5* pathogenic germline mutations. (DOCX)

S1 File Supplemental methods. Statistical analysis for the S1 and S2 Tables.
(DOCX)
