# Supplementary material for: ARMC5 mutations in familial and sporadic primary bilateral macronodular adrenal hyperplasia
Source: PLoS One. 2018 Jan 25;13(1):e0191602. doi: 10.1371/journal.pone.0191602 (PMC5784932; doi:10.1371/journal.pone.0191602)
Supplement: S1 Fig — F1-I-2, F1-II-3 and F1-III-1 exhibited the ARMC5 point mutation c.1855C>T as indicated in panels A, B and C. F1-I-1, F1-II-2, F1-II-4 and F1-III-3 did not exhibit any ARMC5 mutation in the sequences presented in panels D, E, F and G. (PDF) [file pone.0191602.s001.pdf]

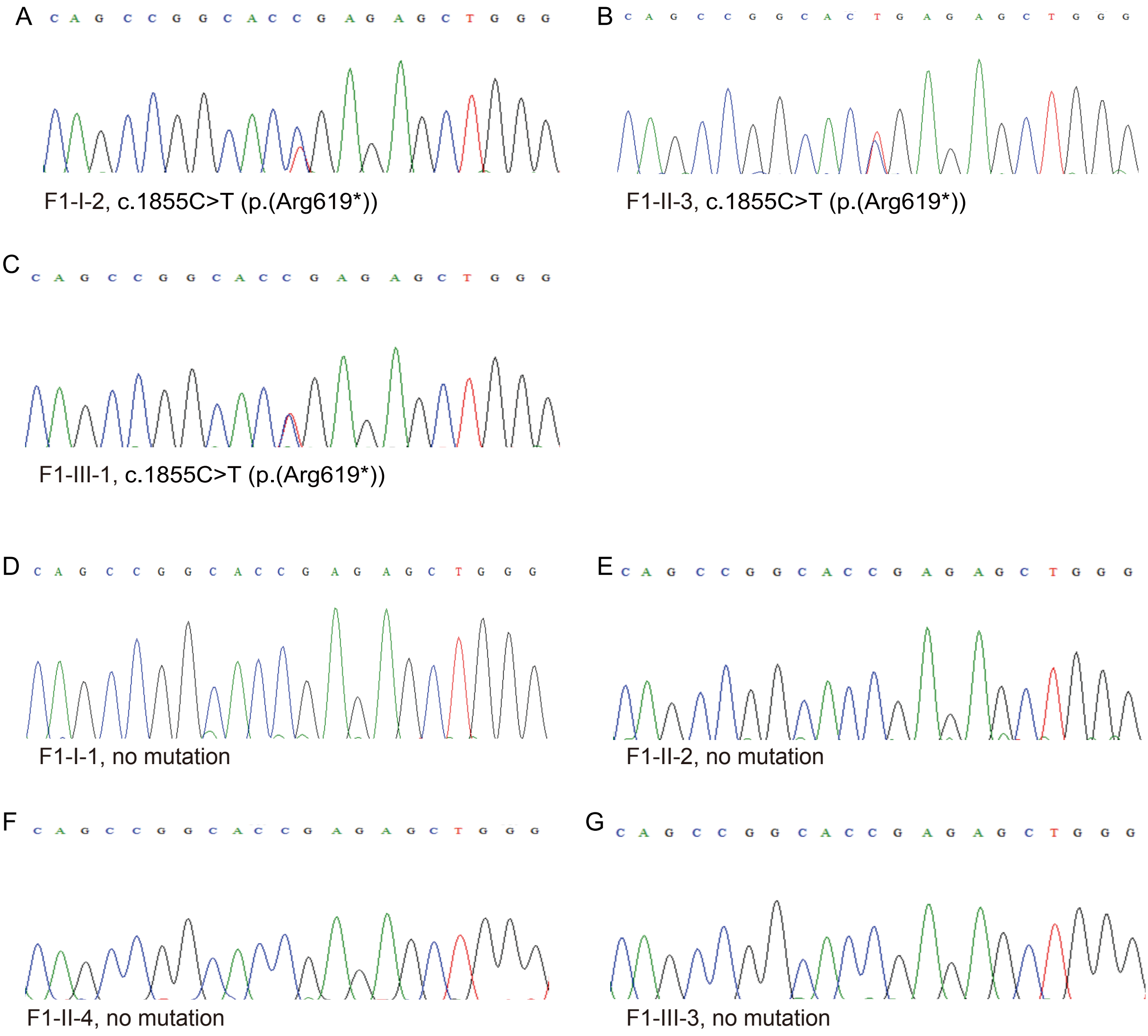

Supplemental figure 1
