# Supplementary figures and images for: ARMC5 mutations in familial and sporadic primary bilateral macronodular adrenal hyperplasia
Source: PLoS One. 2018 Jan 25;13(1):e0191602. doi: 10.1371/journal.pone.0191602 (PMC5784932; doi:10.1371/journal.pone.0191602)

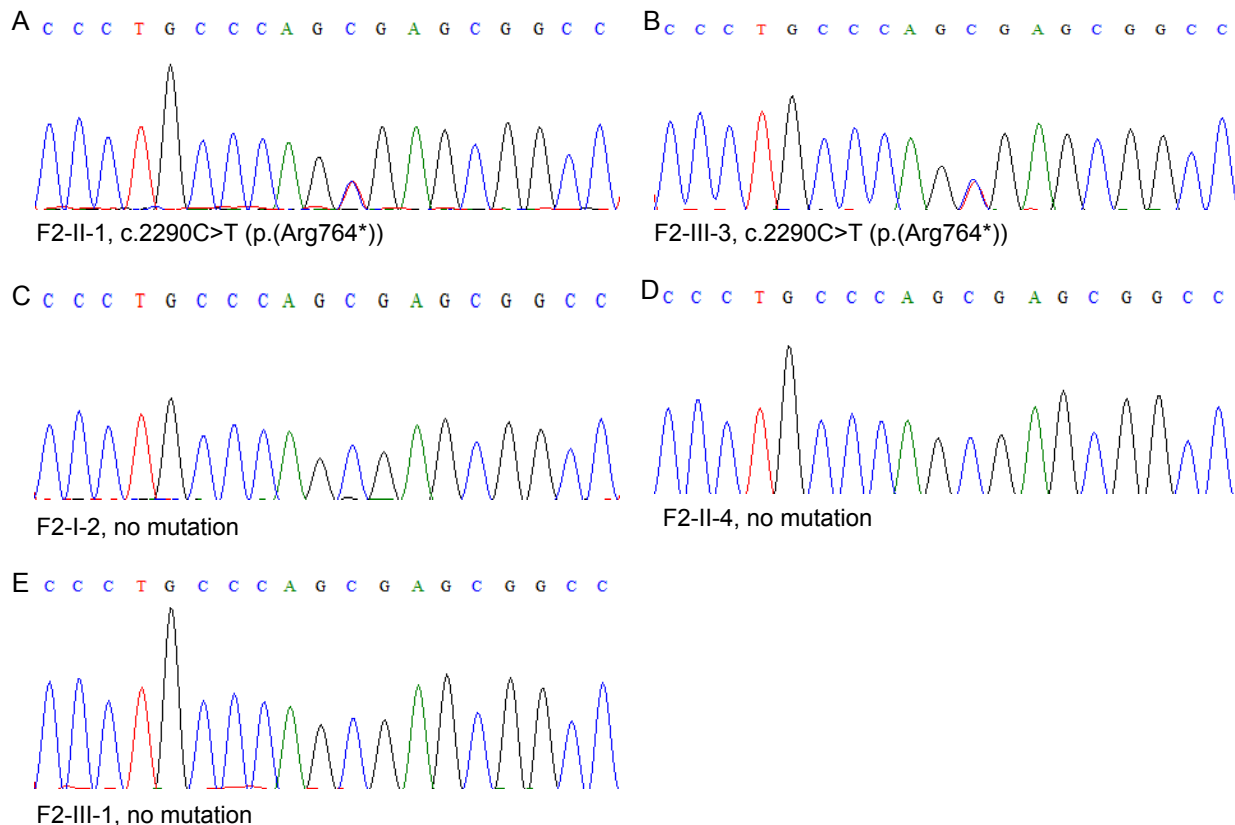

Supplemental figure 2

Supplement: S2 Fig — F2-II-1 and F1-II-3 exhibited the ARMC5 point mutation c.2290C>T as indicated in panels A and B. F2-I-2, F2-II-4 and F2-III-1 did not exhibie any ARMC5 mutation in the sequences presented in panels C, D and E. (PDF) [file pone.0191602.s002.pdf]

A

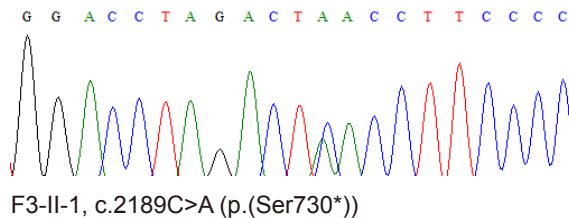

B

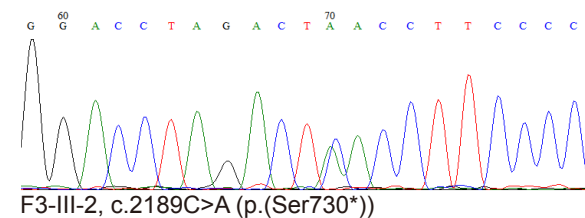

C

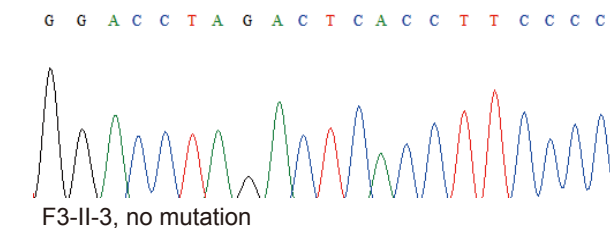

D

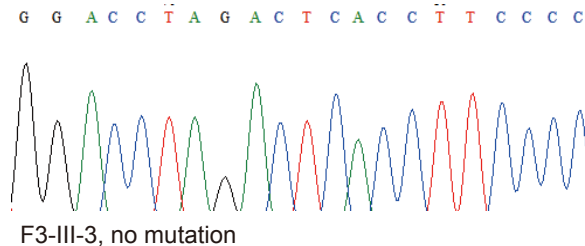

E

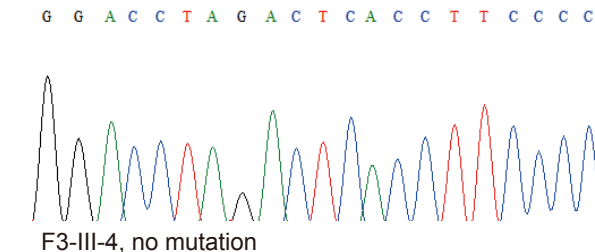

Supplemental figure 3

Supplement: S3 Fig — F3-II-1 and F3-III-2 exhibited the ARMC5 point mutation c.2189C>T as indicated in panels A and B. F3-II-3, F3-III-3 and F3-III-4 did not exhibit any ARMC5 mutation in the sequences presented in panels C, D and E. (PDF) [file pone.0191602.s003.pdf]

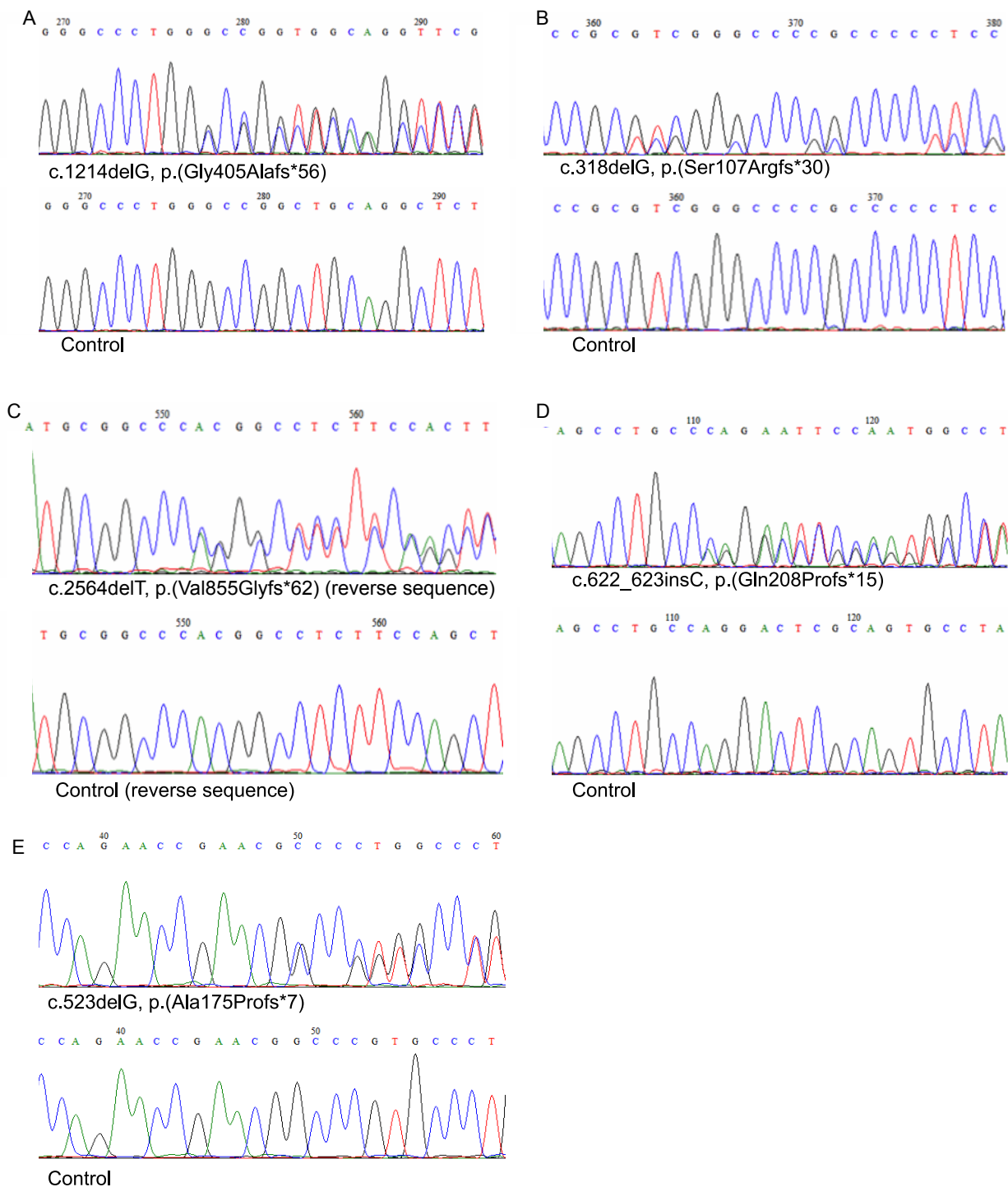

Supplemental figure 4

Supplement: S4 Fig — A, ARMC5 frameshift mutation in P-3: c1214delG, p.(Gly405Alafs*56). B, ARMC5 frameshift mutation in P-6: c.318delG, p.(Ser107Argfs*30). C, ARMC5 frameshift mutation in P-8: c.2564delT, p.(Val855Glyfs*62). This mutation is indicated in the antisense sequencing graph. D, ARMC5 frameshift mutation in P-15: c.622_623insC, p.(Gln208Profs*15). E, ARMC5 frameshift mutation in P-22: p.(Ala175Profs*7). (PDF) [file pone.0191602.s004.pdf]

A

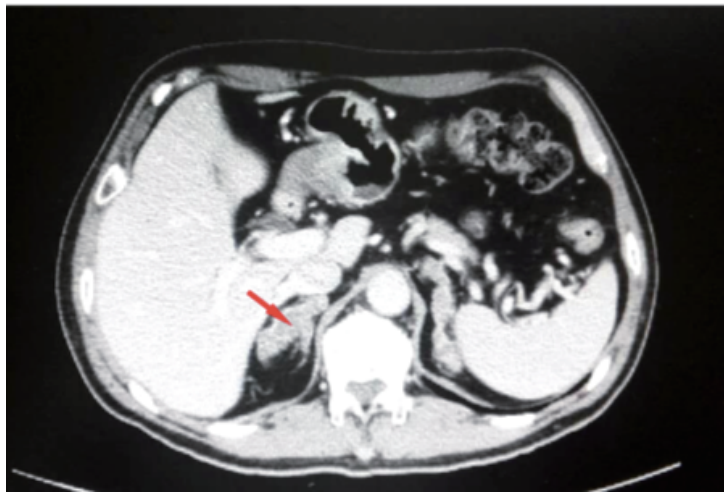

B

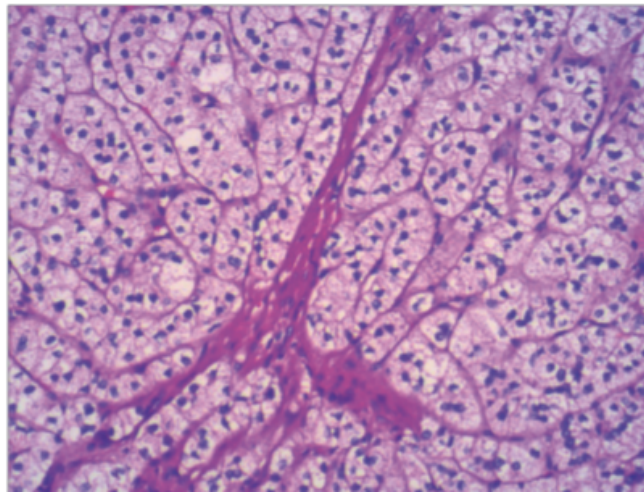

Supplemental figure 5

Supplement: S5 Fig — A. Multiple adrenal nodules were detected in the CT image. The red arrow indicates the right adrenal gland that was surgically resected. B. Histologically, the resected adrenal gland comprised primarily clear-type cells that were rich in lipid. (PDF) [file pone.0191602.s005.pdf]

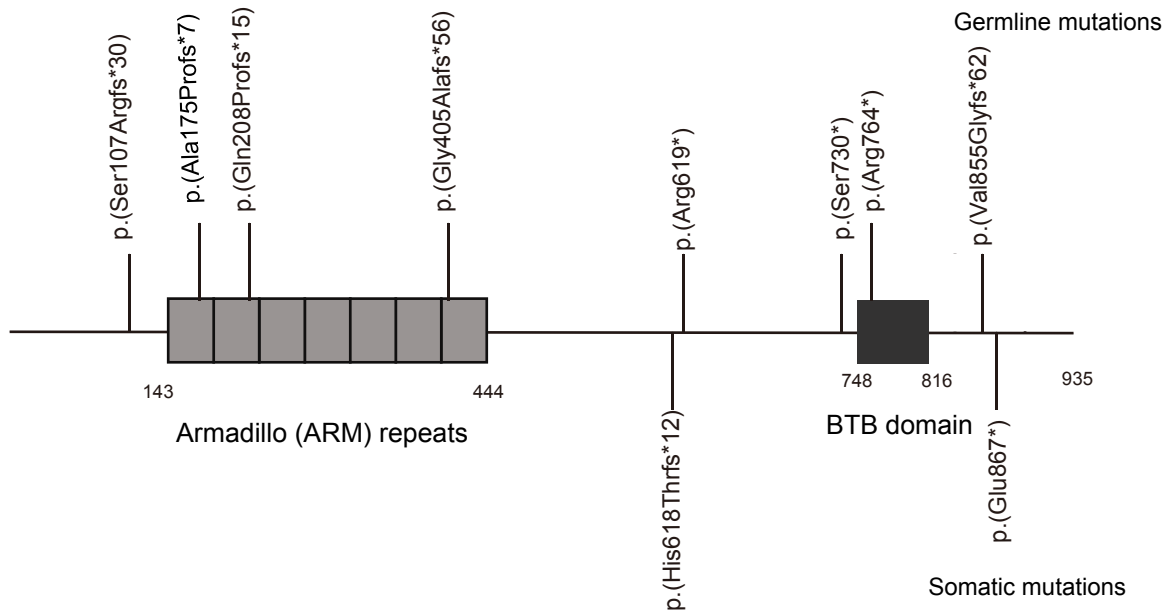

Supplemental figure 6

Supplement: S6 Fig — The ARMC5 protein contains 935 amino acids. The light gray squares indicate the Armadillo repeats, whereas the black square indicates the BTB/POZ domain. The germline mutations are indicated at the upper side of the peptide structure. The somatic mutations are presented under the peptide structure. (PDF) [file pone.0191602.s006.pdf]

A

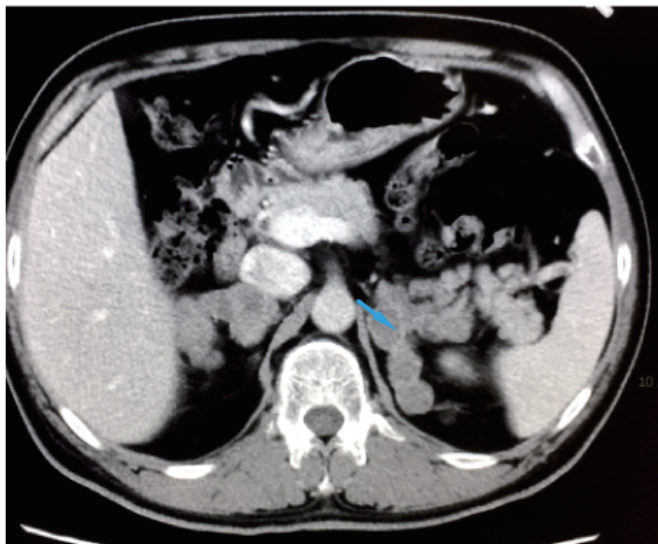

B

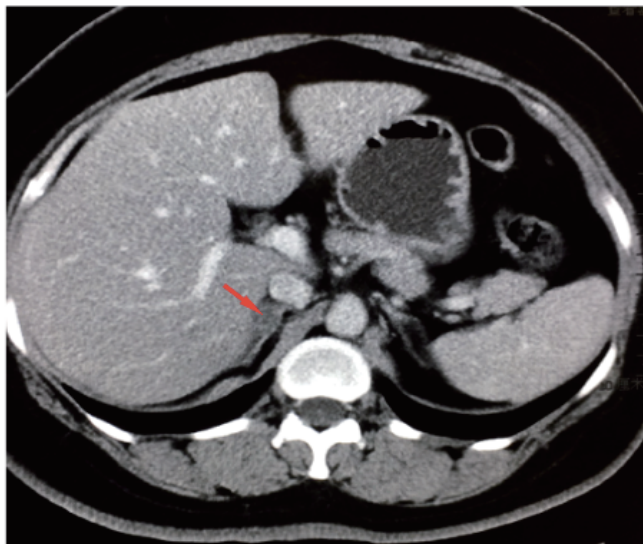

C

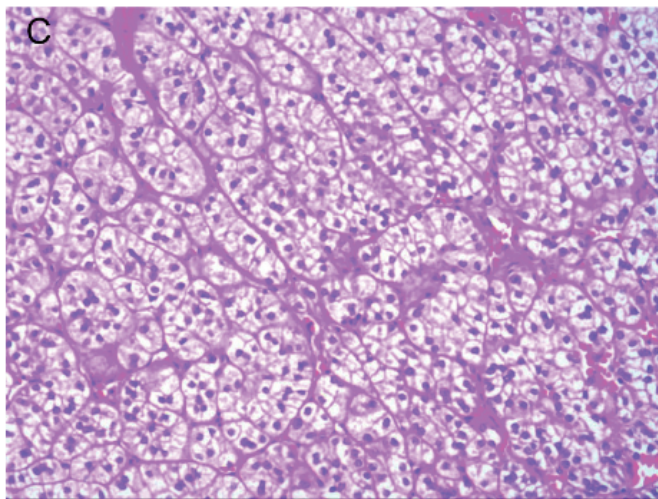

D

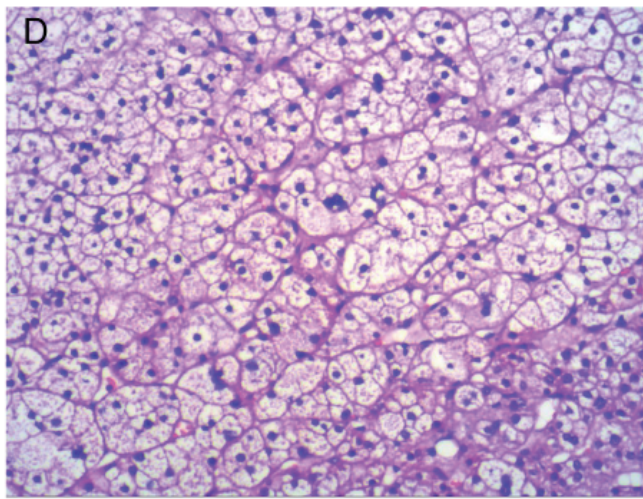

Supplemental figure 7

Supplement: S7 Fig — A. The CT image of the adrenal glands of F1-II-5. The blue arrow indicates the left adrenal gland, which contained multiple nodules and was later resected by surgery. B. The CT image of the adrenal glands of F1-II-4. The red arrow indicates the only nodule on the right adrenal gland. C. The histological image of the resected adrenal nodules of F1-II-5. D. The histological image of the resected adrenal nodule of F1-II-4. (PDF) [file pone.0191602.s007.pdf]

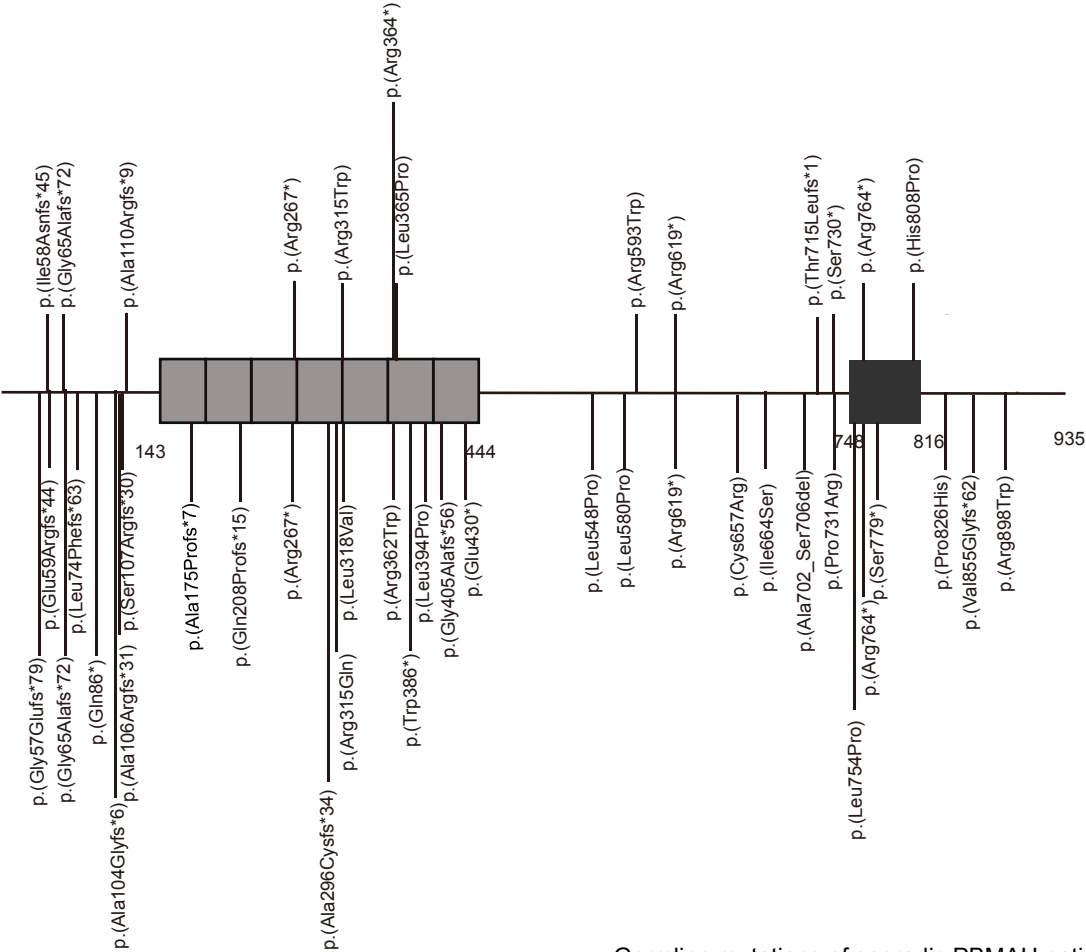

Supplemental figure 8

Supplement: S8 Fig — The ARMC5 protein contains 935 amino acids. The light gray squares indicate the Armadillo repeats, whereas the black square indicates the BTB/POZ domain. The germline mutations of PBMAH families are indicated at the upper side of the peptide structure. The germline mutations of PBMAH sporadic patients are indicated under the peptide structure. (PDF) [file pone.0191602.s008.pdf]
