# Supplementary material for: ARMC5 mutations in familial and sporadic primary bilateral macronodular adrenal hyperplasia
Source: PLoS One. 2018 Jan 25;13(1):e0191602. doi: 10.1371/journal.pone.0191602 (PMC5784932; doi:10.1371/journal.pone.0191602)
Supplement: S1 Table — (DOCX) [file pone.0191602.s009.docx]

Supplemental table 1. Clinical data of sporadic PBMAH patients without *ARMC5* pathogenic germline mutations

| PBMAH patients | Gender | Age (y) | Morning cortisol (ug/dl) | Morning ACTH (PG/ml) | Adrenal masses and the size of unilateral adrenal masses | diabetes | HT | PA | Cushing’s syndrome | Adrenal surgery |
| --- | --- | --- | --- | --- | --- | --- | --- | --- | --- | --- |
| P-1 | F | 56 | 17.38 | 21.14 | Bilateral adrenal nodules, the largest nodule in right side is 1 cm diameter | No | Yes | Yes | Yes, subclinical | No |
| P-2 | M | 49 | 20.85 | <1 | L: multiple nodules, 11.05 cm^3^;  R: multiple nodules, 8.69 cm^3^ | No | Yes | No | Yes, overt | Bilateral |
| P-4 | M | 58 | 13.87 | <1 | L: multiple nodules, 7.20 cm^3^;  R: multiple nodules, 4.94 cm^3^ | Yes | Yes | Yes | Yes, subclinical | Unilateral |
| P-5 | M | 63 | 17.15 | 20.06 | L: multiple nodules, 19.43 cm^3^;  R: multiple nodules, 3.30 cm^3^ | IGT | Yes | No | Yes, subclinical | Left |
| P-7 | M | 44 | 12.26 | <1 | Bilateral multiple nodules | No | Yes | No | Yes, subclinical | Unilateral |
| P-9 | M | 56 | 19.29 | 3.83 | L: multiple nodules, 0.88 cm^3^;  R: multiple nodules, 1.07 cm^3^ | Yes | Yes | No | Yes, subclinical | No |
| P-10 | F | 71 | 22.41 | 26.71 | L: multiple nodules, 3.71 cm^3^;  R: multiple nodules, 0.36 cm^3^ | No | Yes | Yes | Yes, subclinical | Bilateral |
| P-11 | M | 56 | 27.65 | 30.79 | L: multiple nodules, 2.51 cm^3^;  R: multiple nodules, 3.63 cm^3^ | Yes | Yes | Yes | Yes, subclinical | Right |
| P-12 | M | 43 | 26.23 | 1.96 | L: multiple nodules, 9.42 cm^3^;  R: multiple nodules, 39.25 cm^3^ | IFG | Yes | No | Yes, overt | Left |
| P-13 | F | 43 | 13.86 | 80.41 | Bilateral multiple nodules | IGT | Yes | No | Yes, overt | Left |
| P-14 | M | 56 | 16.48 | 7.18 | Bilateral multiple nodules | IFG | Yes | No | Yes, subclinical | No |
| P-16 | M | 63 | 24.6 | 44.48 | Bilateral multiple nodules | Yes | Yes | Yes | Yes, subclinical | No |
| P-17 | M | 55 | 17.83 | 78.02 | L: a nodule, 0.61 cm^3^;  R: a nodule, 0.18 cm^3^ | Yes | Yes | Yes | Yes, subclinical | No |
| P-18 | M | 52 | 21.81 | <1 | L: multiple nodules, 41.61 cm^3^;  R: multiple nodules, 33.49 cm^3^ | Yes | Yes | No | Yes, overt | No |
| P-19 | M | 69 | 29.35 | 27.66 | bilateral multiple nodules | No | Yes | No | Yes, subclinical | No |
| P-20 | F | 51 | 16.49 | 32.12 | L: a nodule, 1.43 cm^3^;  R: a nodule, 0.12 cm^3^ | Yes | Yes | Yes | Yes, subclinical | right |
| P-21 | F | 47 | 31.39 | <1 | L: multiple nodules, 6.02 cm^3^;  R: multiple nodules, 0.63 cm^3^ | No | Yes | No | Yes, overt | left |
| P-23 | M | 57 | 30.32 | 16.74 | L: a nodule, 0.57 cm^3^;  R: a nodule, 2.00 cm^3^ | No | Yes | No | Yes, subclinical | No |
