# Supplementary material for: ARMC5 mutations in familial and sporadic primary bilateral macronodular adrenal hyperplasia
Source: PLoS One. 2018 Jan 25;13(1):e0191602. doi: 10.1371/journal.pone.0191602 (PMC5784932; doi:10.1371/journal.pone.0191602)
Supplement: S2 Table — (DOCX) [file pone.0191602.s010.docx]

Supplemental table 2. Comparison between sporadic PBMAH patients with and without *ARMC5* pathogenic germline mutations

| Characteristics | *ARMC5* mutated ^a^ | Without *ARMC5* mutation ^a^ | *P value* ^c^ |
| --- | --- | --- | --- |
| Number of patients | 5 | 18 |  |
| Age (years) | 60 [53-65] | 56 [49-59] | 0.37 |
| Gender (F/M) | 2/3 | 5/13 | 0.62 |
| Overt CS/subclinical CS | 4/1 | 5/13 | 0.056 |
| Hypertension (%) | 100 | 100 | 1 |
| Glucose metabolism (%) |  |  | 0.67 |
| diabetes | 3 | 7 |  |
| IGT/IFG | 1 | 4 |  |
| Normal glucose metabolism | 1 | 7 |  |
| BMI (kg/m^2) | 27.5 [23.3-30.4] | 27.0 [25.2-28.2] | 0.74 |
| UFC (ug/24h) | 872 [433-4997] | 900 [603-1209] | 0.72 |
| Morning serum cortisol (ug/dl) | 21.2 [15.7-41.8] | 20.1 [16.5-26.6] | 0.80 |
| Late night cortisol (ug/dl) | 9.8 [4.6-30.1] | 6.5 [3.8-12.7] | 0.49 |
| Serum cortisol after LDDST (ug/dl) | 17.0 [3.2-35.5] | 3.1 [2.2-16.4] | 0.19 |
