# Supplementary material for: ARMC5 mutations in familial and sporadic primary bilateral macronodular adrenal hyperplasia
Source: PLoS One. 2018 Jan 25;13(1):e0191602. doi: 10.1371/journal.pone.0191602 (PMC5784932; doi:10.1371/journal.pone.0191602)
Supplement: S1 File — Statistical analysis for the S1 and S2 Tables. (DOCX) [file pone.0191602.s011.docx]

**Supplemental methods**

Statistical analysis for S1 and S2 Tables

The Mann Whitney test was applied to compare the quantitative data of *ARMC5*-mutated PBMAH patients and those of *ARMC5* wildtype PBMAH patients. The Chi-square test or Fisher’s exact test was used to compare the qualitative data. Quantitative data are presented as median [25% percentile – 75% percentile]. Qualitative data are presented as numbers.
